# Multi-criteria decision analysis approach on parametric optimization of abrasive waterjet pocket milling in Ti-6Al-4V alloy

**DOI:** 10.1038/s41598-025-26719-1

**Published:** 2025-11-28

**Authors:** K. L. Naresh Raj, S. Jayavelu, M. Siva Kumar, Ajay Kumar, B. Swarna

**Affiliations:** 1 Department of Mechanical Engineering, Vel Tech Rangarajan Dr. Sagunthala R&D Institute of Science and Technology, Avadi, 600062 India; 2 Department of Mechanical Engineering, SRM TRP Engineering College, Tiruchirapalli, 621105 India; 3 Department of Mechanical Engineering, School of Engineering, Faculty of Science, Technology & Architecture, Manipal University Jaipur, Jaipur, 303007 India; 4 Department of Biosciences, Saveetha School of Engineering, Saveetha Institute of Medical and Technical Sciences, Chennai, 602105 India

**Keywords:** AWJ, Pocket milling, PSO, MFO, GWO, WOA, MOORA, CODAS, Engineering, Materials science, Mathematics and computing

## Abstract

The multi criteria decision analysis (MCDA) approach was used to optimise Abrasive waterjet (AWJ) pocket milling process parameters to examine the machinability of Ti-6Al-4 V alloy and the experiments were done by the L_32_ Taguchi array. Abrasive mesh (AM), waterjet pressure (WP), and traverse speed (TS) were chosen for this study, and material removal rate (MRR), depth of cut (DC), undercut (UC), and surface roughness (SR) were studied. Furthermore, the multiparametric ANOVA was used to determine the statistical significance of milling parameters, and the spatial pattern of output response parameter values was statistically assessed to support process parameter selection. Finally, regression models were developed based on milling parameters and output. To estimate the optimum level of input parameters for multi-objective optimization criteria on milling, particle swarm optimization (PSO), moth-flame optimization (MFO), grey wolf optimization (GWO), and whale optimization algorithm (WOA) have been combined with the combinative distance-based assessment (CODAS) methodology and multi-objective optimization on the basis of ratio analysis (MOORA). Weights for output were assigned using the objective-based entropy weight-age approach. The entropy weights for DC, UC, SR, and MRR were 0.3255, 0.2312, 0.1837, and 0.2596, respectively. The MOORA method predicts the best ideal parameters and based on the assessment scores MFO algorithm outperformed the others in maximising DC and MRR while reducing UC and SRR.

## Introduction

In conventional machining, pocket milling, which involves removing material arbitrarily, is one of the most difficult forms of machining. This is due to the fact that tool failure is the most typical constraint, which drives up component prices due to the fact that the tool that broke must be replaced. Researchers have resorted to using the AWJ milling process, which is able to do rough stage milling, in order to circumvent this obstacle. It has been demonstrated that AWJ is capable of providing a high-quality machine tool for the processing of materials that are free of chipping, component faults, and thermal stress^1^. High-velocity abrasive waterjets cause material loss in both cutting and deformation wear modes^2^. The WP, AM, TS, AFS, and SoD parameters are the ones that are utilised in AWJ operations rather frequently^3^. In addition, the variables that are employed in milling processes, such as WP, TS, and AM, still have a limited amount of flexibility, which means that the values of these components cannot be increased or decreased. These are the most important characteristics that, when combined, contribute to the higher energy generation that the jet achieves when processing materials.

Researchers have employed AWJ to mill pockets^4,5^ by adjusting input parameters such as WP, TS, and AM. Additionally, several types of abrasives have also been used; however, only few of these studies have showed optimization in the AWJ pocket milling process^6^. It is of the utmost importance to ascertain the appropriate values for the process parameters in order to achieve an improvement in the efficiency of the processing. When the parameter settings are optimised, high-quality machining may be accomplished. Numerous evolutionary algorithms, such as the adaptive wavelet network^7^, cuckoo^8^, PSO^9^, MFO^10^, and others have been used by researchers in their works in an effort to determine the parameters that should be considered ideal or optimal. It was discovered that the evolutionary algorithms were successful in parametric study when the AWJ procedures were being carried out^11^. Previous AWJ milling studies provided valuable insights into the influence of waterjet pressure, abrasive type and traverse speed on performance metrics such as depth of cut and material removal rate; however, most of these works reported only single‑objective optimization and lacked comprehensive comparisons of multiple metaheuristic algorithms. Moreover, many prior experiments did not fully describe their methodologies or report measurement uncertainties, limiting reproducibility.

In spite of the fact that AWJ machining is applied often, the potential of milling processes is not being fully utilised. In addition, in-depth study on the AWJ milled pockets has not yet been carried out. As a consequence of this, the purpose of this investigation is to investigate the effect of process parameters on the performance of AWJ pocket milling by making use of soft computing strategies such as the PSO, MFO, GWO, and WOA algorithms. In addition to this, the effectiveness of the algorithms is evaluated by employing the CODAS and MOORA multi-criteria decision analysis approaches. Although AWJ milling has been studied, optimization combining multi‑criteria decision analysis with evolutionary algorithms for Ti‑6Al‑4 V pocket milling has not been systematically explored. The ranges of WP (200–400 MPa), AM (80–150 mesh) and TS (2500–4000 mm/min) used in this work were selected based on the capabilities of the AWJ machine, supplier recommendations for garnet abrasives and preliminary trials on Ti‑6Al‑4 V, ensuring that the selected levels cover practical operating conditions.

## Materials and methods

### Experimental setup

**Fig. 1:**
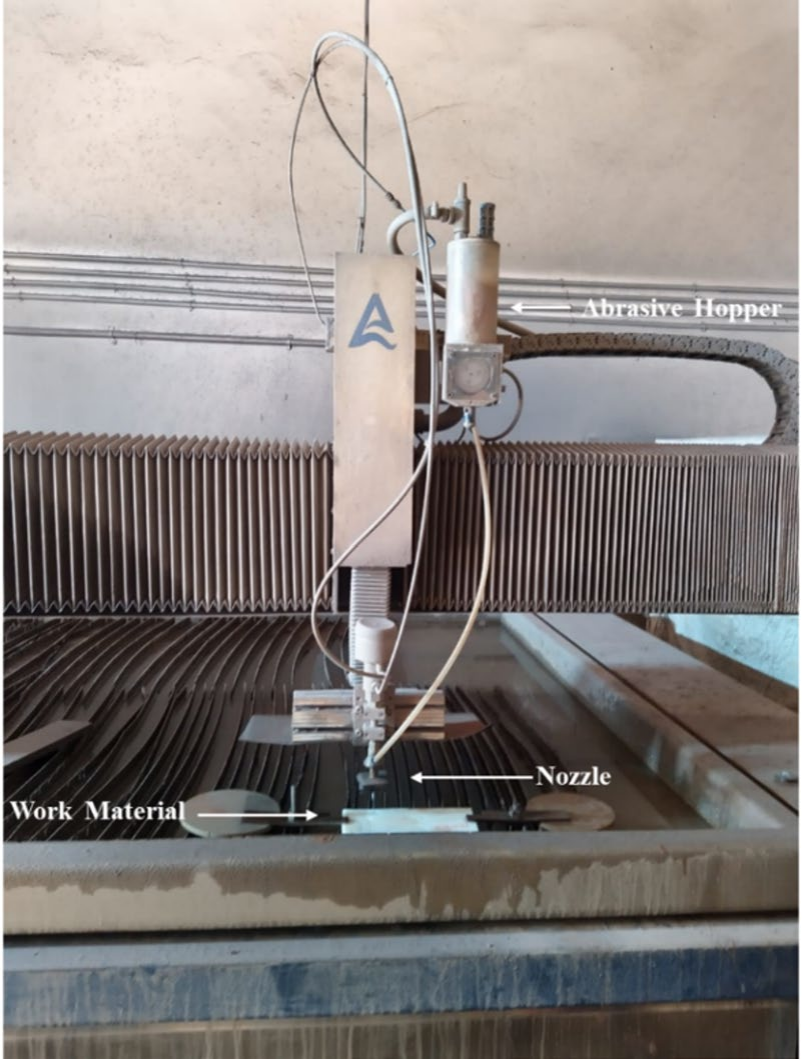
Experimental setup.

The work material for pocket milling operations in this investigation was Ti-6Al-4 V titanium alloy. The target material has dimensions of 150 × 150 × 8 mm. Milling trials were conducted on an AWJ machine tool (model G3020) with a capacity of 400 MPa (60 HP pump). The experimental setup for milling operations is shown in Fig. 1. In the target material, a 20 × 20 mm square pocket was milled.  Table 1 displays the AWJ milling parameters and their levels. AM, TS, and WP were the variable parameters in this work, and the abrasive used was garnet with a flow rate of 350 g/min. All experiments were conducted in accordance with the institutional and laboratory safety standards for high-pressure abrasive waterjet machining systems, including the use of appropriate personal protective equipment, adherence to operational pressure limits, and implementation of emergency and waste disposal protocols.

**Table 1:** AWJ milling settings.

Parameters	Levels
AM, #	80, 100
TS, mm/min	2500, 3000, 3500, 4000
WP, MPa	125, 150, 175, 200

**Fig. 2:**
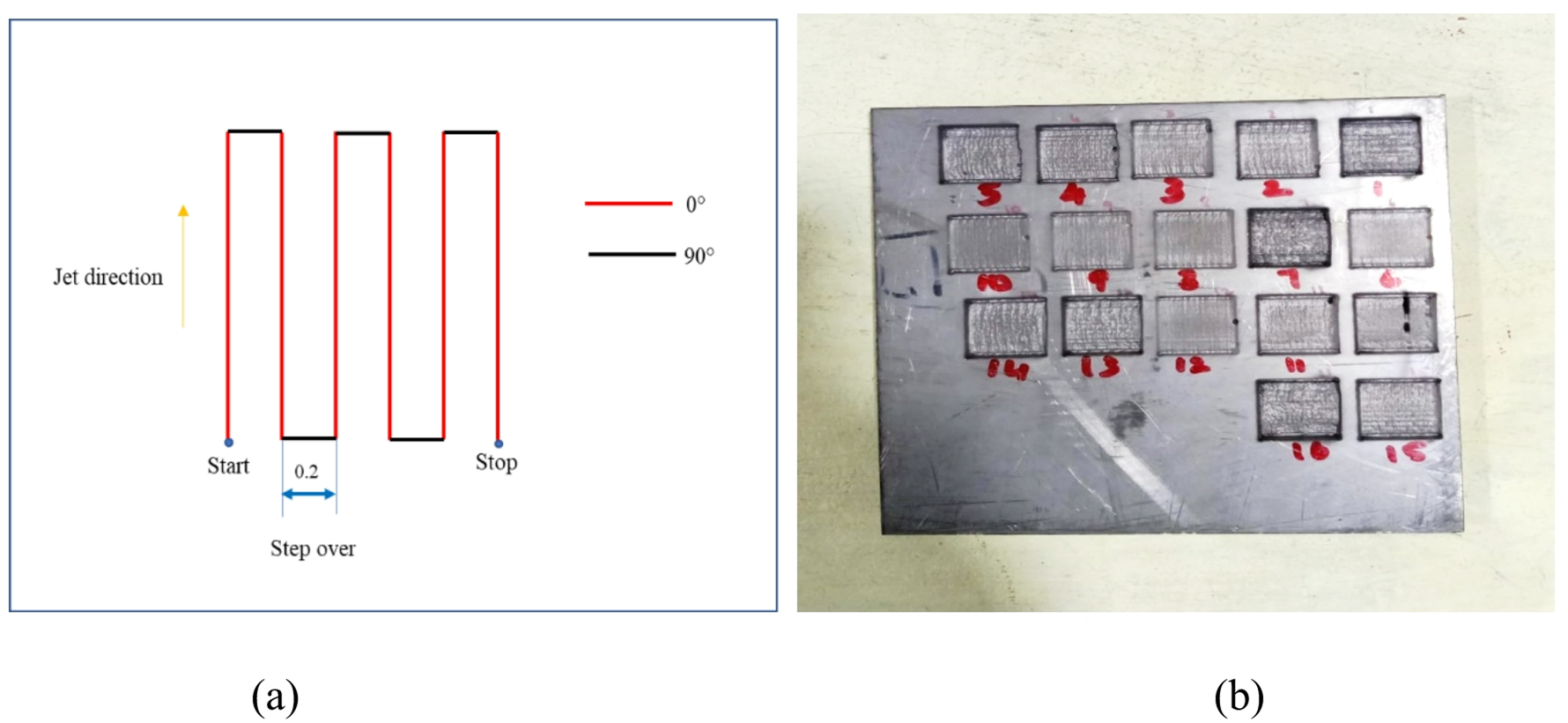
AWJ milling setup (**a**) raster tool path (**b**) sample milled blind pockets.

The raster path that was programmed on the water jet machine tool was used to create the blind pockets. The raster path depicted in Fig. 2(a) was interpreted as a straight cut route down which the jet travels. After completing each pass, the jet rotated through an angle of ninety degrees before continuing linearly in accordance with the pre-specified step across a distance of 0.2 mm. After that, it completed yet another round of ninety degrees and moved on to the subsequent straight cut. This process was done several times until the necessary area had been milled. The AWJ milled samples of the Ti-6Al-4 V alloy are displayed in Fig. 2(b). Milling trials were carried out in accordance with the L_32_ model by design of experiments with regard to the variable parameters that were specified. The characteristics of the pocket that were examined were the DC, UC, SR, and MRR. The Taguchi L32 orthogonal array was chosen because it efficiently explores three factors at four levels with a reduced number of experiments, providing balanced coverage of the design space and enabling robust estimates of factor effects.

### Measurement of output responses

To measure DC and UC, a digital microscope with a magnification up to 220x was utilised. Furthermore, the Digital Microscope is used to measure the UC that occurred at the square pocket’s edges. UC is an unfavourable characteristic that frequently appears in AWJ machined pockets. Each experimental trial was replicated three times to account for variability, and average values are reported. The measurement uncertainties for DC, UC and SR were ± 5 μm, ± 10 μm and ± 0.1 μm respectively, based on the instrument accuracies provided by the manufacturers.

The MRR was calculated by ratio of volume difference to milling time using Eq. 1. A weighing scale was used to determine the weight (Make: Eagle, Model: PKT-10 C).

1 $$\:MRR,\:g/\text{min}=\:\frac{\:{Material\:weight}_{before\:milling}-{Material\:weight}_{after\:milling}\:}{time\:taken\:}\:$$

Surface roughness was assessed using a contact roughness tester (Surfcom 1400G) with a 0.8 mm cut off length and 3.2 mm traverse length.

### MCDM methods

#### CODAS

CODAS (combinative distance-based assessment) is a multi-criteria decision-making (MCDM)^12^ method that ranks alternatives based on beneficial and non-beneficial criteria using ideal and anti-ideal solutions^13^. The methodology for CODAS is:

Developing the initial decision matrix

$$\:X={\left[{x}_{ij}\right]}_{n\times\:m}=\left[\begin{array}{cccc}{x}_{11}&\:{x}_{12}&\:\cdots\:&\:{x}_{1m}\\\:{x}_{21}&\:{x}_{22}&\:\cdots\:&\:{x}_{2m}\\\: \vdots &\: \vdots &\: \vdots &\: \vdots \\\:{x}_{n1}&\:{x}_{n2}&\:\cdots\:&\:{x}_{nm}\end{array}\right],\text{n}\text{\:and\:}\text{m}\text{\:are\:number\:of\:alternatives\:and\:criteria\:}.$$

Normalize decision matrix

$$\:{n}_{ij}=\left\{\begin{array}{lll}\frac{{x}_{ij}}{\underset{i}{\text{m}\text{a}\text{x}}\:{x}_{ij}}&\:\text{\:if\:}j\in\:{N}_{b}&\:\\\:\frac{\underset{i}{\text{m}\text{i}\text{n}}\:{x}_{ij}}{{x}_{ij}}&\:\text{\:if\:}j\in\:{N}_{c}&\:{\text{}\text{N}}_{\text{b}}\text{\:and\:}{\text{N}}_{\text{c}}\text{\:represent\:benefit\:and\:cost\:criteria\:}\end{array}\right.$$

Weighted normalized decision matrix

$$\:{\text{r}}_{ij}={\text{w}}_{j}{\text{n}}_{ij}$$

Determine the negative ideal solution points

$$\:\begin{aligned}\text{n}\text{s}& ={\left[{\text{n}\text{s}}_{j}\right]}_{1\times\:\text{m}}\\\:{\text{}\text{n}\text{s}}_{\text{j}}&=\text{m}\text{i}\text{n}{\text{r}}_{ij}\end{aligned}$$

Calculate the Euclidean and Taxicab distances of alternatives from the negative ideal solution

$$\:\begin{aligned} {E}_{i} & =\sqrt{\sum\:_{j=1}^{m}\:\:{\left({r}_{ij}-n{s}_{j}\right)}^{2}}\\ {T}_{i} & =\sum\:_{j=1}^{m}\:\:\left|{r}_{ij}-n{s}_{j}\right|\end{aligned}$$

Construct the relative assessment matrix

$$\:\begin{aligned}\:\text{R}\text{a}& ={\left[{\text{h}}_{ik}\right]}_{\text{n}\text{x}\text{n}}\\ {\text{}\text{h}}_{ik} &=\left({\text{E}}_{l}-{\text{E}}_{k}\right)+\left(\psi\:\left({\text{E}}_{i}-{\text{E}}_{k}\right)\times\:\left({\text{T}}_{i}-{\text{T}}_{k}\right)\right)\end{aligned}$$

Calculate the assessment score and rank the alternatives

$$\:{\text{H}}_{i}=\sum\:_{k=1}^{n}\:{\text{}\text{h}}_{ik}$$

#### MOORA

MOORA (Multi-Objective Optimization on the basis of Ratio Analysis) is a popular MCDM method^14,15^. The methodology for MOORA is:

Initial decision matrix

$$\:X=\left[\begin{array}{ccccc}{x}_{11}&\:{x}_{12}&\:\dots\:&\:\dots\:&\:{x}_{1n}\\\:{x}_{21}&\:{x}_{22}&\:\dots\:&\:\dots\:&\:{x}_{2n}\\\:\dots\:&\:\dots\:&\:\dots\:&\:\dots\:&\:\dots\:\\\:\dots\:&\:\dots\:&\:\dots\:&\:\dots\:&\:\dots\:\\\:{x}_{m1}&\:{x}_{m2}&\:\dots\:&\:\dots\:&\:{x}_{mn}\end{array}\right]\text{n}\text{\:and\:}\text{m}\text{\:are\:number\:of\:attributes\:and\:alternatives\:}$$

Normalize decision matrix

$$\:{\stackrel{\prime }{x}}_{ij}^{\text{*}}={x}_{ij}/{\left[\sum\:_{i=1}^{m}\:\:{x}_{ij}^{2}\right]}^{1/2}(j=\text{1,2},\dots\:,n)$$

Estimation of Assessment values

$$\:{y}_{i}=\sum\:_{j=1}^{g}\:{w}_{j}{x}_{ij}^{\text{*}}-\sum\:_{j=g+1}^{n}\:{w}_{j}{x}_{ij}^{\text{*}}(j=\text{1,2},\dots\:,n)$$

### Metaheuristic methods

#### Particle swarm optimization (PSO)

PSO is a swarm intelligence-based optimization algorithm inspired by the social behavior of birds flocking and fish schooling^16^. Each particle adjusts its position using both its own experience and the swarm’s best solution^17,18^.

**Algorithm 1:**
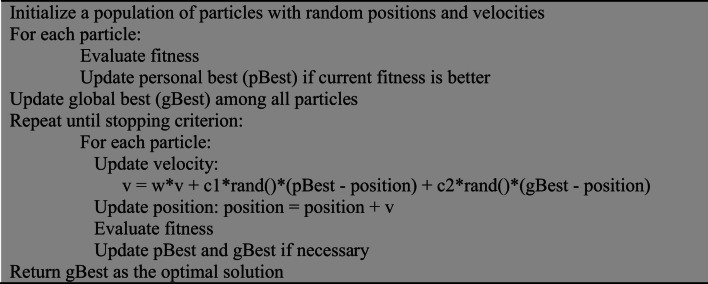
Pseudocode for PSO.

#### Moth-flame optimization (MFO)

MFO is inspired by the navigation behavior of moths around flames, where candidate solutions (moths) update their positions relative to flames to balance exploration and exploitation^19,20^.

**Algorithm 2:**
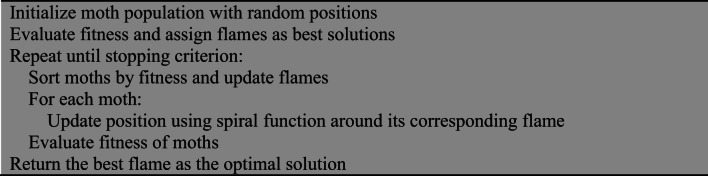
Pseudocode for MFO.

#### Grey Wolf Optimization (GWO)

GWO mimics the leadership hierarchy and hunting strategy of grey wolves, using alpha, beta, and delta wolves to guide the search while other wolves update positions accordingly^21,22^.

**Algorithm 3:**
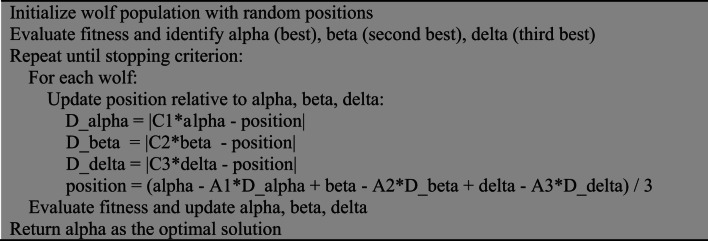
Pseudocode for GWO.

#### Whale optimization algorithm (WOA)

WOA simulates the bubble-net hunting behavior of humpback whales, where solutions encircle prey and update their positions using shrinking encircling and spiral movements^23,24^.

**Algorithm 4:**
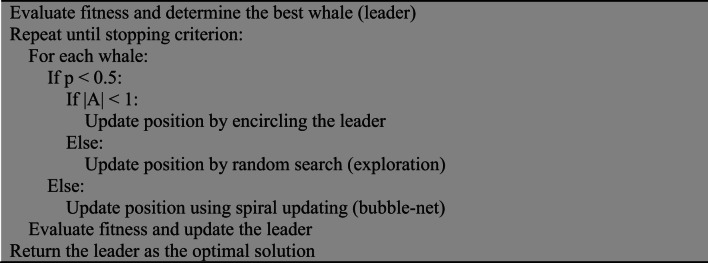
Pseudocode for WOA.

The various parameters used in the metaheuristics are reported in Table 2. In this study both CODAS and MOORA were applied as multi‑criteria decision analysis methods to provide cross‑validation and enhance confidence in the ranking of alternative parameter combinations. Algorithm control parameters such as population size, inertia weights and learning coefficients were selected based on standard values reported in the literature to ensure fair comparisons across PSO, MFO, GWO and WOA. In addition to numerical metrics, statistical tests (ANOVA) were performed on the IGD and SP metrics and confirmed that the observed differences among algorithms were significant at the 95% confidence level.

**Table 2:** PSO, MFO, GWO, WOA parameter values.

PSO algorithm		MFO algorithm		GWO algorithm		WOA algorithm	
Parameter	Value	Parameter	Value	Parameter	Value	Parameter	Value
No. of iterations	100	No. of iterations	100	No. of Iteration	100	No. of iteration	100
Learning factors (C1 & C2)	2 & 2	Position of moth close to the flame	− 1 to − 2	Scale Facto	2 to 0	Position of whale	Value of AM, TS and WP
Inertia weight	0.6	Update mechanism	Logarithmic spiral	Population Size	30	Number of dimensions involved in defining the position of whale	3
Particle size	30	No. of moths	30			Number of whales	30
						Position of prey	Best value of AM, TS and WP
						Fitness of whale	Value of DC, UC. SR and MRR

## Results and discussion

To conduct pocket milling experiments on the target Titanium (Ti-6Al-4 V) alloy, multiple combinations of process control settings based on the L_32_ orthogonal array were configured on the AWJ machine. Furthermore, as described in Sect. 2.2, the DC, UC, SR, and MRR were recorded. Table 3 displays the experimental observations and output responses.

**Table 3:** Experimental design and output response.

Exp. no.	AM (#)	TS (mm/min)	WP (MPa)	DC (mm)	UC (mm)	SR (µm)	MRR (mm^3^/min)
1	80	2500	125	0.376	0.643	1.91	1.057
2	80	2500	150	0.783	1.152	2.14	1.853
3	80	2500	175	1.596	1.779	4.68	2.669
4	80	2500	200	2.448	2.734	7.66	4.326
5	80	3000	125	0.246	0.47	1.92	0.718
6	80	3000	150	0.647	0.87	2.08	1.655
7	80	3000	175	1.238	1.992	3.24	2.979
8	80	3000	200	2.003	2.711	6.22	4.191
9	80	3500	125	0.264	0.384	1.99	0.918
10	80	3500	150	0.489	0.881	2.21	1.344
11	80	3500	175	0.955	1.443	3.27	2.388
12	80	3500	200	1.611	2.316	6.14	3.835
13	80	4000	125	0.149	0.436	2.05	0.886
14	80	4000	150	0.343	0.996	2.41	1.213
15	80	4000	175	0.783	1.887	2.44	2.277
16	80	4000	200	1.265	2.768	7.24	4.064
17	100	2500	125	0.492	0.604	1.89	0.864
18	100	2500	150	0.91	1.197	2.87	0.904
19	100	2500	175	1.715	2.103	5.98	2.909
20	100	2500	200	2.394	3.155	7.87	4.918
21	100	3000	125	0.246	0.615	1.62	1.021
22	100	3000	150	0.649	1.219	2.16	1.379
23	100	3000	175	1.26	1.969	3.77	2.689
24	100	3000	200	2.02	2.707	7.39	4.639
25	100	3500	125	0.238	0.526	2.18	0.833
26	100	3500	150	0.522	1.029	2.45	1.224
27	100	3500	175	0.992	1.521	3.65	1.284
28	100	3500	200	1.618	2.45	3.97	4.286
29	100	4000	125	0.194	0.514	2.11	0.480
30	100	4000	150	0.47	0.951	2.51	1.391
31	100	4000	175	0.859	2.17	3.78	2.700
32	100	4000	200	1.297	3.076	4.61	4.145

The ANOVA was performed on the DC, UC, SR, and MRR responses using Minitab software and the results are shown in Table 4. The P-value from ANOVA results of the linear, square and 2-way interaction models for DC and SR was less than 0.05. As a result, all of the input factors used in this study were statistically significant. And the P-values for the linear and square models were less than 0.05 for UC and MRR, whereas the P-value for the 2-way interaction model for UC was greater than 0.05, indicating that the parameters have no effect on the algorithm’s performance. Specifically, waterjet pressure (WP) is the dominant factor for DC, SR and MRR because it sets the jet kinetic energy; travelling speed (TS) controls exposure time and therefore strongly affects DC, SR and MRR (with non-linear effects captured by TS²); abrasive mesh (AM) shows notable influence for DC and UC (edge-cutting behaviour) but limited effect on SR and MRR within the tested mesh range. Significant quadratic terms (WP², TS²) and the TS×WP interaction (for DC and SR) reflect non-linear and coupled effects of pressure and speed.

**Table 4:** ANOVA for various responses.

Source	DF	DC		UC		SR		MRR	
		Adj SS	P-value	Adj SS	P-value	Adj SS	P-value	Adj SS	P-value
Model	8	13.7607	0.000	23.1551	0.000	109.096	0.000	57.0934	0.000
Linear	3	12.9610	0.000	22.5661	0.000	91.724	0.000	53.3519	0.000
AM	1	0.0145	0.025	0.1717	0.024	0.046	0.758	0.0157	0.712
TS	1	1.9541	0.000	0.0860	0.100	4.254	0.006	0.6487	0.025
WP	1	10.9925	0.000	22.3084	0.000	87.424	0.000	52.6875	0.000
Square	2	0.2556	0.000	0.5649	0.001	11.115	0.000	3.3809	0.000
TS*TS	1	0.0362	0.001	0.2930	0.004	1.945	0.054	0.0507	0.509
WP*WP	1	0.2195	0.000	0.2720	0.006	9.170	0.000	3.3302	0.000
2-Way interaction	3	0.5441	0.000	0.0240	0.844	6.257	0.013	0.3606	0.381
AM*TS	1	0.0000	0.908	0.0007	0.874	1.038	0.151	0.0032	0.868
AM*WP	1	0.0012	0.501	0.0194	0.424	0.366	0.387	0.2479	0.151
TS*WP	1	0.5429	0.000	0.0038	0.721	4.853	0.004	0.1096	0.334
Error	23	0.0579		0.6746		10.801		2.5853	
Total	31	13.8187		23.8297		119.898		59.6788	

**Fig. 3:**
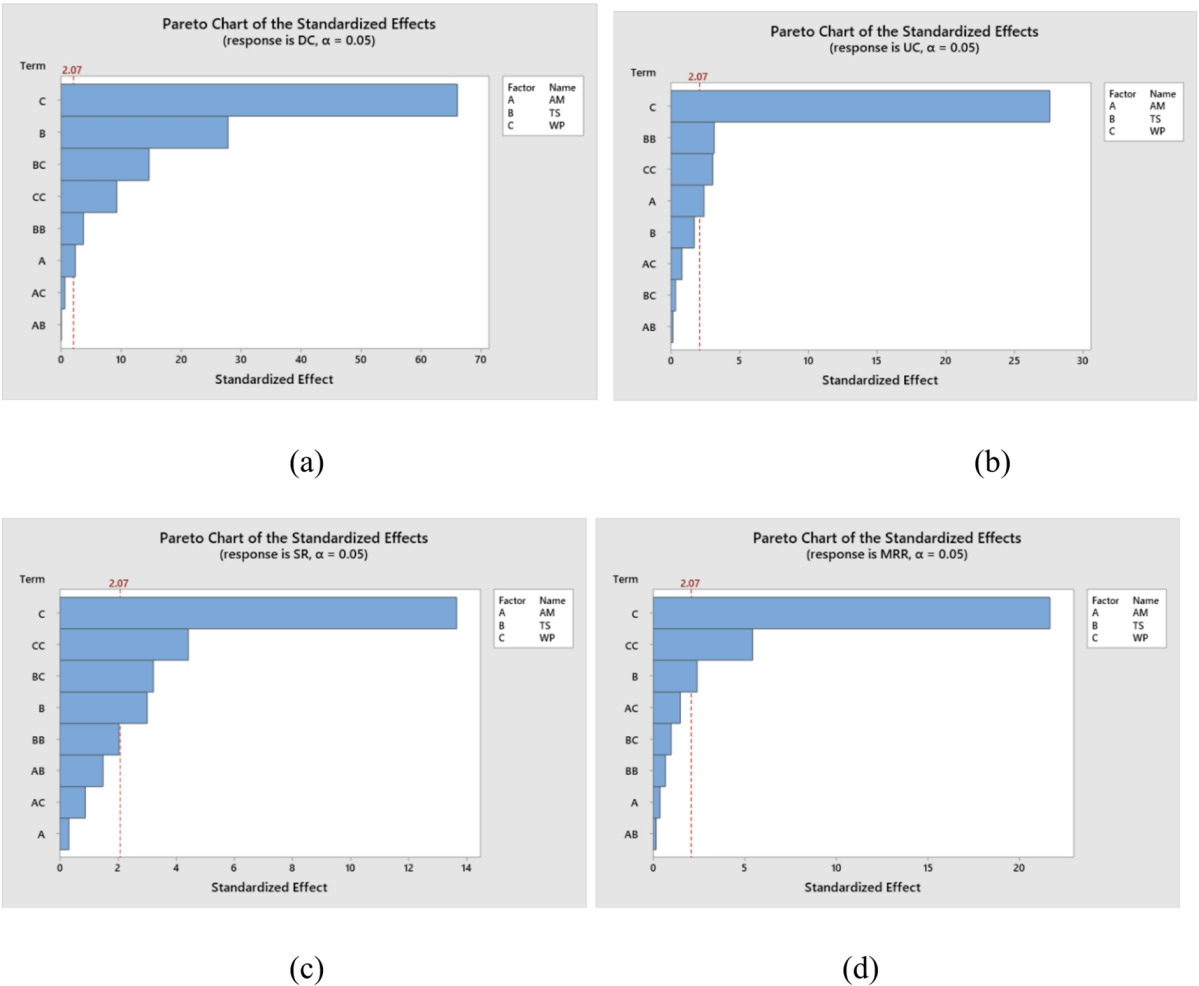
Pareto analysis of (**a**) DC, (**b**) UC, (**c**) SR, (**d**) MRR.

**Fig. 4:**
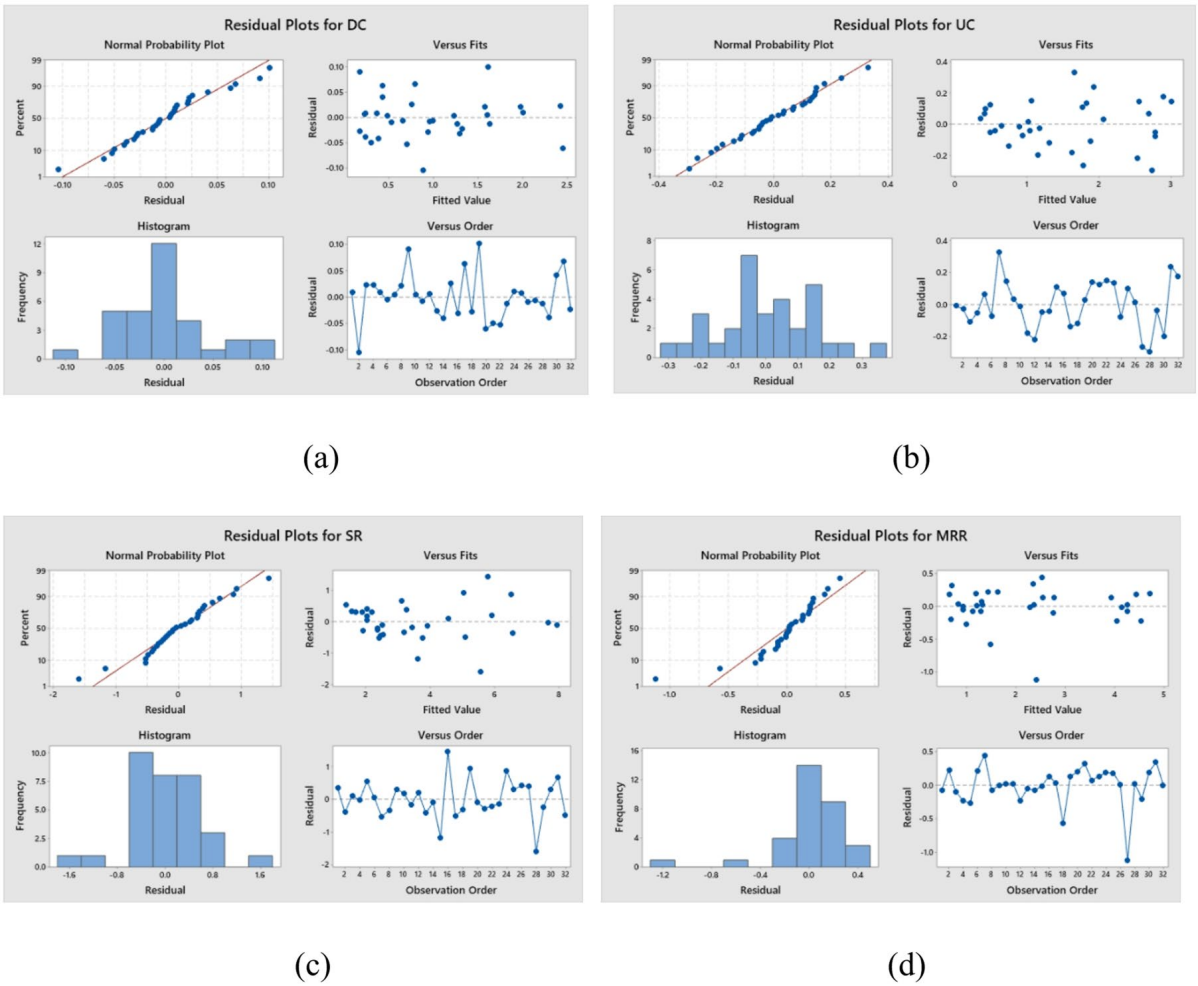
Residual Plots for (**a**) DC, (**b**) UC, (**c**) SR, (**d**) MRR.

Pareto charts for investigating the influence of milling variables on DC, UC, SR, and MRR are shown in Fig. 3a-d. Figure 3a, c and d show that the input parameters, specifically WP and TS, had the greatest influence on the DC, SR, and MRR values. In addition, for UC value, WP and TS were the most influential, while AM was the least influential. Figure 4a-d shows response parameter spatial distribution. Figure 4 shows experimental and predicted milling response residuals. This residual plot was created using the Taguchi L_32_ orthogonal array and AWJ pocket milling experimental data such as DC, UC, SR, and MRR. Using residual plots, the difference between experimental and predicted residuals was examined.

The normal probability graphs illustrate that each milling performance’s residues established a structural pattern (Fig. 4). The residual vs. fitted values plot demonstrates that the residues were randomly distributed demonstrating a correlation that is not linear and containing no variances in the data. Residue plot histograms showed varying residue frequencies for each AWJ milling operation. In each experiment, residual vs. data order plots confirm residue distribution. Each experiment’s residue is determined by a unique arrangement of process parameters and their interactions under milling conditions. The findings demonstrate that the model that was suggested for the AWJ residue analysis applies to milling performance variables. According to these statistics, the input parameters were adjusted correctly to minimise UC and SR while improving DC and MRR.

Furthermore, using Minitab quadratic type MLRMs for DC, UC, SR, and MRR were created based on the relationship between the AWJ pocket milling process parameters and the output response characteristics. The MLRM for DC, UC, SR, and MRR are as follows:

$$\begin{aligned} \text{D}\text{C}&=-1.91+0.00625\left(AM\right)+0.000055\left(TS\right)+0.00695\left(WP\right) \\ & \quad +0.000000{\left(TS\right)}^{2}+0.000133{\left(WP\right)}^{2}-\:\:\:0.000000\left(AM\right)\left(TS\right)\\ & \quad -0.000022\left(AM\right)\left(WP\right)-0.000008\left(TS\right)\left(WP\right) \end{aligned}$$

$$\begin{aligned} \text{U}\text{C}&=5.44-0.0042\left(AM\right)-0.002617\left(TS\right)-0.0283\left(WP\right) \\ & \quad +0.000000{\left(TS\right)}^{2}+0.000147{\left(WP\right)}^{2}-0.000001\left(AM\right)\left(TS\right)\\ & \quad +0.000088\left(AM\right)\left(WP\right)+0.000001\left(TS\right)\left(WP\right) \end{aligned}$$

$$\begin{aligned} \text{S}\text{R}&=-0.3+0.171\left(AM\right)-0.00011\left(TS\right)-0.1038\left(WP\right)\\ & \quad +0.000001{\left(TS\right)}^{2}+0.000857{\left(WP\right)}^{2}-0.000032\left(AM\right)\left(TS\right)\\ & \quad -0.000382\left(AM\right)\left(WP\right)-0.000025\left(TS\right)\left(WP\right) \end{aligned}$$

$$\begin{aligned} \text{M}\text{R}\text{R}& =13.82-0.0592\left(AM\right)-0.00084\left(TS\right)-0.1380\left(WP\right)\\ & \quad +0.000000{\left(TS\right)}^{2}+0.000516{\left(WP\right)}^{2}+\:0.000002\left(AM\right)\left(TS\right)\\ & \quad+0.000315\left(AM\right)\left(WP\right)-0.000004\left(TS\right)\left(WP\right) \end{aligned}$$

**Fig. 5:**
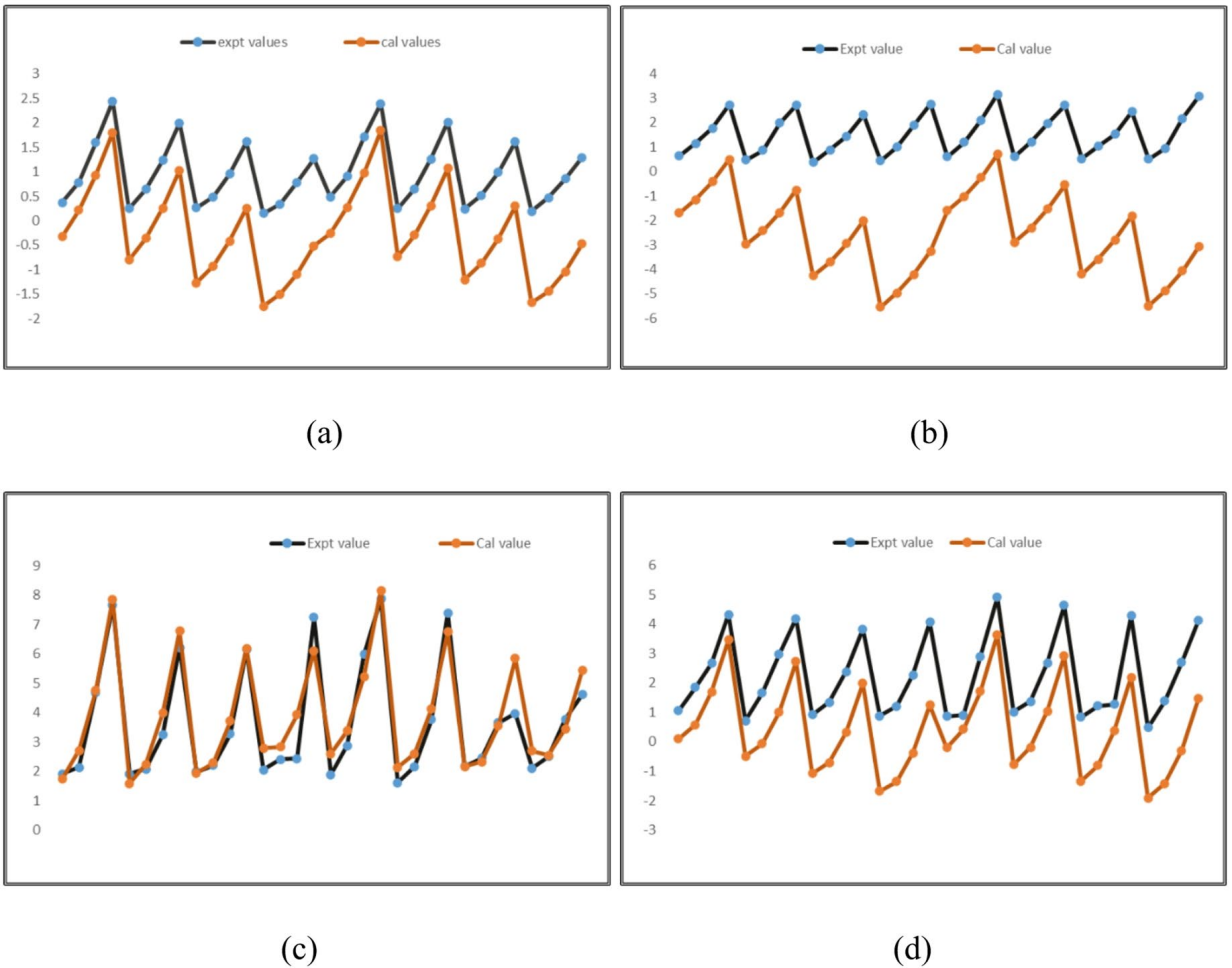
Experimental and calculated values of response (**a**) DC, (**b**) UC, (**c**) SR, (**d**) MRR.

Based on the statistical research discussed above, the process of developing the MLRM for the response, only the relevant factors and their interactions were taken into consideration individually. The process of comparing the calculated values to the experimental results is shown in Fig. 5. The developed quadratic regression models for DC, UC, SR and MRR exhibited high coefficients of determination (R² values exceeding 0.95 for all responses), demonstrating strong predictive capability which are presented in Table 5.

**Table 5:** Performance of regression models.

Metrics	DC	UC	SR	MRR
R^2^	0.9958	0.9717	0.9099	0.9567
RMSE	0.0425	0.1452	0.5810	0.2843

PSO, MFO, GWO, and WOA algorithms have been combined with CODAS, a multi-criteria decision analysis tool in this work. The CODAS technique seeks to undertake a multi-criteria selection process based on the highest Euclidean and Taxicab distance in relation to negative ideal solutions. This approach compares the MOORA method to determine the best process parameter for AWJ pocket milling.

**Fig. 6:**
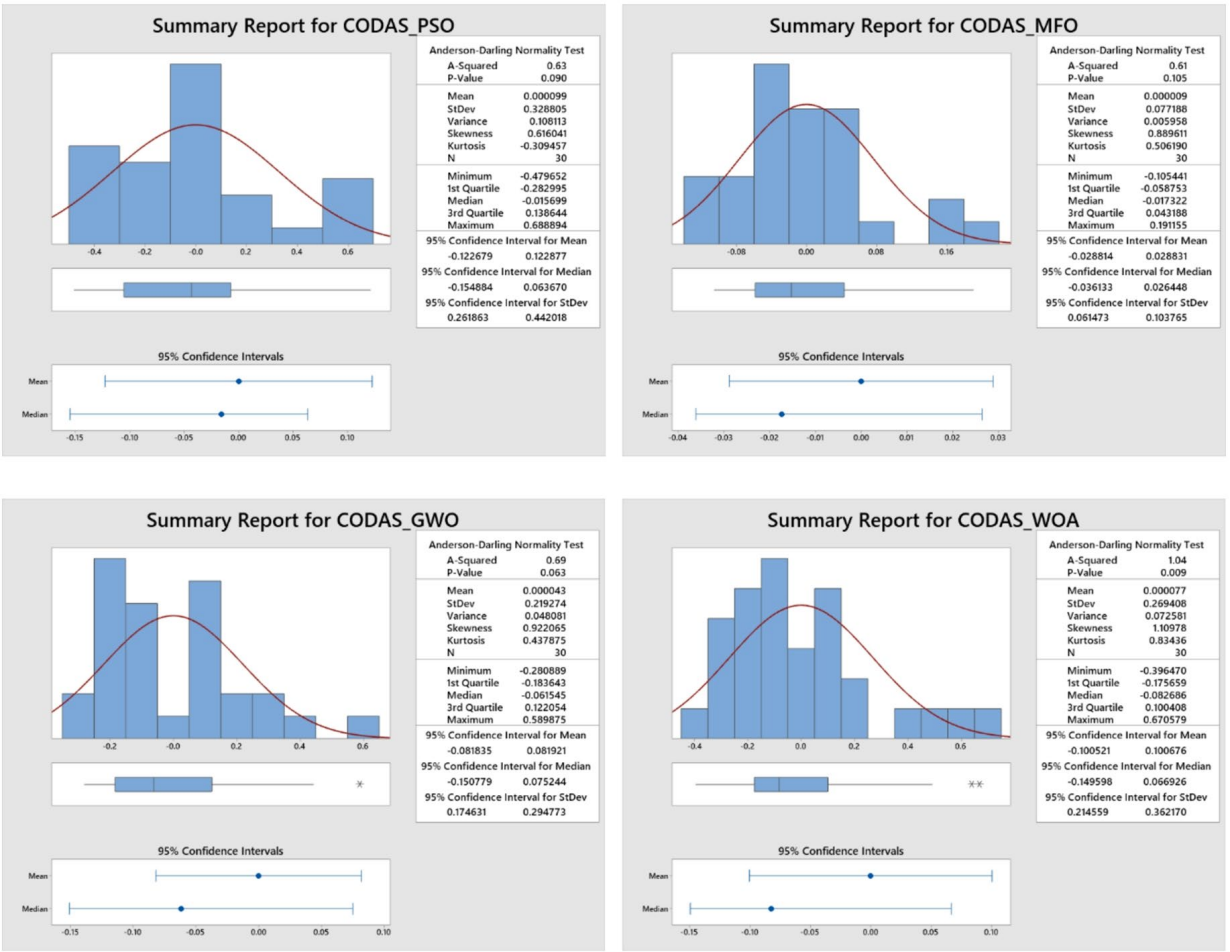
Distribution plot for CODAS.

**Fig. 7:**
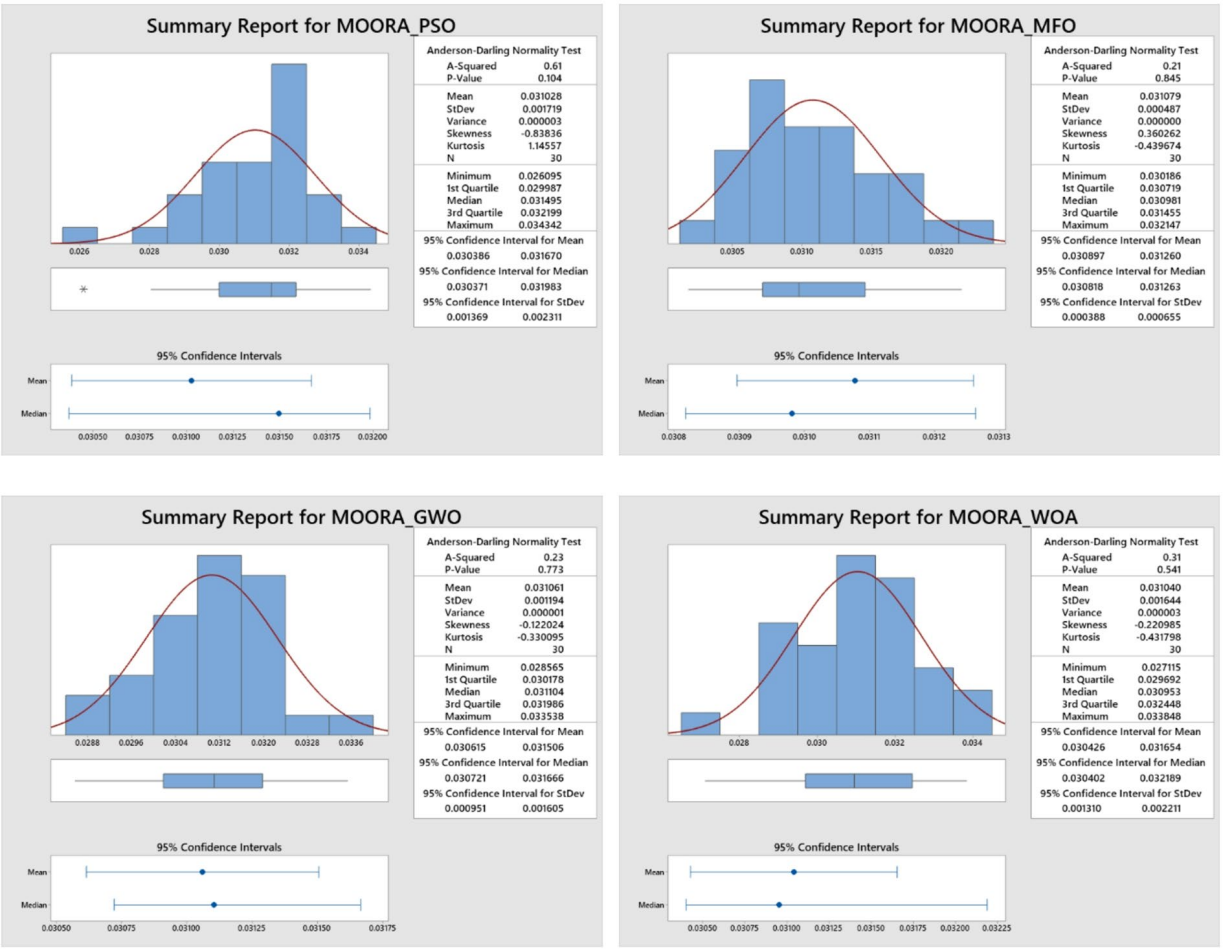
Distribution plot for MOORA.

Figures 6 and 7 depicts a distribution plot of the MCDA approach for the aforementioned algorithm, and it reveals that the spatial distribution of calculation time is normally distributed for both CODAS and MOORA. As a result, the major parameters for the multi criterion decision analysis procedures are properly calibrated in order to achieve a reduction in the amount of time spent on calculations during the AWJ pocket milling procedure. According to these findings, the calibrated major parameters were used for the execution of the CODAS and MOORA procedures in order to create improved pocket characteristics in the AWJ milling process.

Table 6 shows the optimum parameter and its response achieved by performing the aforementioned MCDA method for 30 runs with 100 iterations per run and a population size of 30. The results reveal that the MFO algorithm in the MOORA technique outperformed the other algorithms and the CODAS technique. Furthermore, as indicated by Khalilpourazari et al.^25^, the algorithm’s performance is tested using SP and IGD metrics. Table 7 shows the statistical value (SP &IGD) of the algorithms.

**Table 6:** Best optimum process parameter and its response.

Method	Algorithm	AM	TS	WP	DC	UC	SR	MRR
CODAS	PSO	83.5424	2524.5422	199.2082	2.3746	2.7795	7.5815	4.4941
	MFO	81.4101	2516.5880	199.8982	2.4046	2.7878	7.6746	4.5401
	GWO	97.0046	2509.7635	199.9728	2.4364	2.9708	7.9113	4.6797
	WOA	99.3655	2500.2118	198.4303	2.3923	2.9403	7.7631	4.5670
MOORA	PSO	83.5424	2524.5422	199.2082	2.3746	2.7795	7.5815	4.4941
	**MFO**	**80.3210**	**2515.9316**	**200.0000**	**2.4074**	**2.7800**	**7.6769**	**4.5401**
	GWO	85.1945	2500.7075	199.7060	2.4180	2.8327	7.7421	4.5650
	WOA	90.0876	2512.2664	199.0442	2.3897	2.8538	7.6810	4.5403

**Table 7:** Statistical performance of algorithm.

Method	Algorithm	IGD	SP
CODAS	PSO	0.14988	0.06213
	**MFO**	**0.03994**	**0.01803**
	GWO	0.12153	0.02384
	WOA	0.10735	0.08840
MOORA	PSO	0.15129	0.06119
	**MFO**	**0.03277**	**0.02651**
	GWO	0.12601	0.03929
	WOA	0.10788	0.08667

**Fig. 8:**
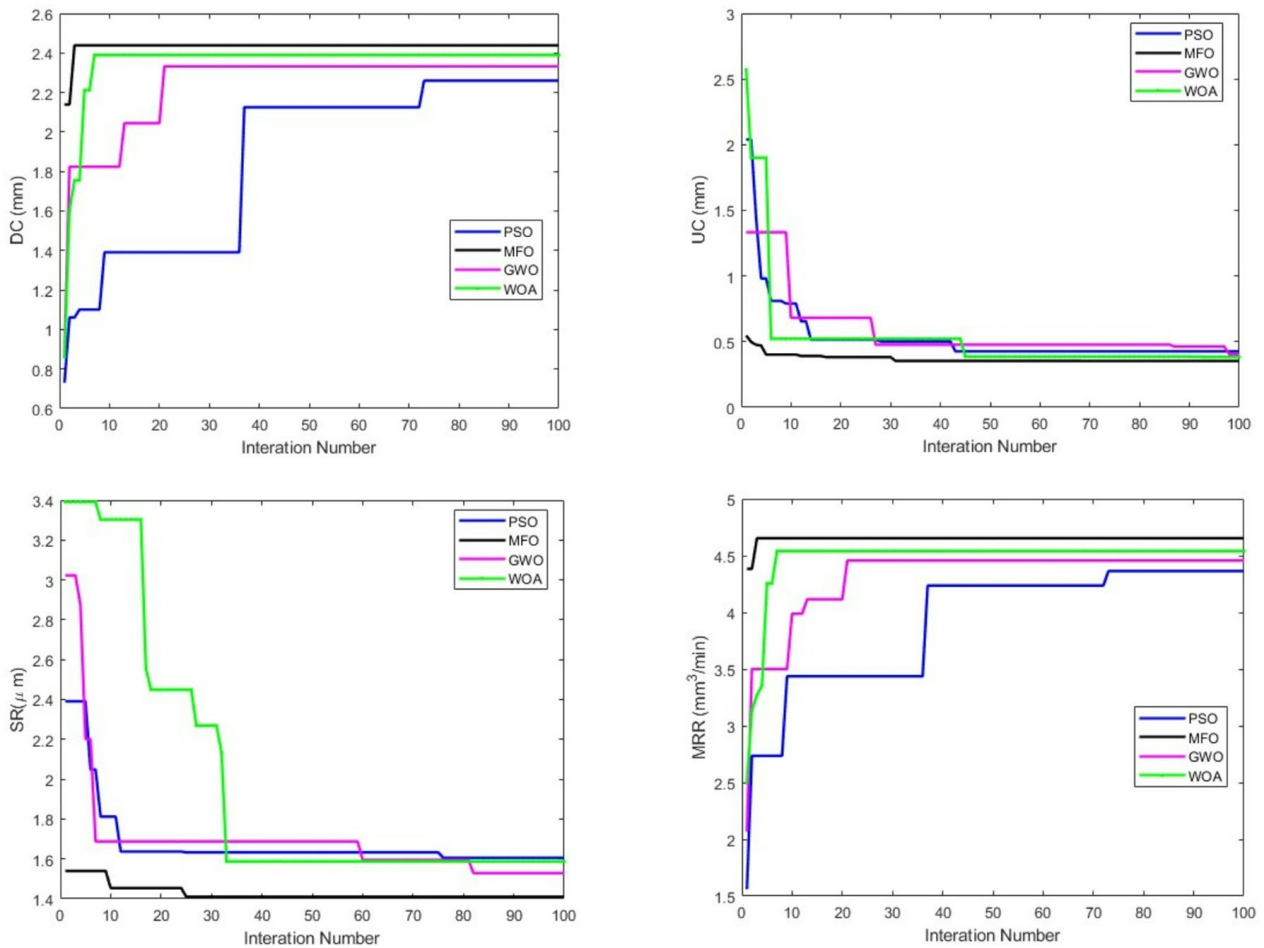
Convergence plot for DC, UC, SR, MRR.

According to Table 7, the value of SP and IGD in MFO is lower than in other. As an outcome, based on the measures utilised, it is understandable that the MFO algorithm excelled other algorithms for the present work. Furthermore, the convergence graphs for the DC, UC, SR, MRR in Fig. 8 demonstrated MFO’s effectiveness. In this work, the convergence of all optimization algorithms (MFO, PSO, WOA, and GWO) was determined based on a fixed iteration count, which was kept identical across all algorithms to ensure a fair comparison. As an outcome of this, it is possible to draw the conclusion that the MFO performed better than the PSO, GWO, and WOA. Moreover, the superior performance of the Moth Flame Optimization (MFO) algorithm in this study can be attributed to its inherent ability to balance exploration and exploitation effectively. MFO simulates the transverse orientation navigation of moths, where candidate solutions (moths) move around flames in a logarithmic spiral path. This dynamic search mechanism enables MFO to explore the global search space efficiently while simultaneously refining promising regions locally, thereby avoiding premature convergence to local optima. In comparison, Particle Swarm Optimization (PSO) tends to converge rapidly but may be trapped in local minima, Whale Optimization Algorithm (WOA) emphasizes exploitation over exploration and may struggle in multimodal landscapes, and Grey Wolf Optimization (GWO) provides a balanced search but often converges slower in highly nonlinear problem spaces. In our experiments on abrasive water jet milling, MFO consistently achieved superior outcomes across all responses—material removal rate, depth of cut, undercut, and surface roughness—demonstrating that its spiral navigation and exploration–exploitation trade-off are particularly effective for the complex, constrained optimization problem considered in this work.

### DC at different AWJ milling parameters

**Fig. 9:**
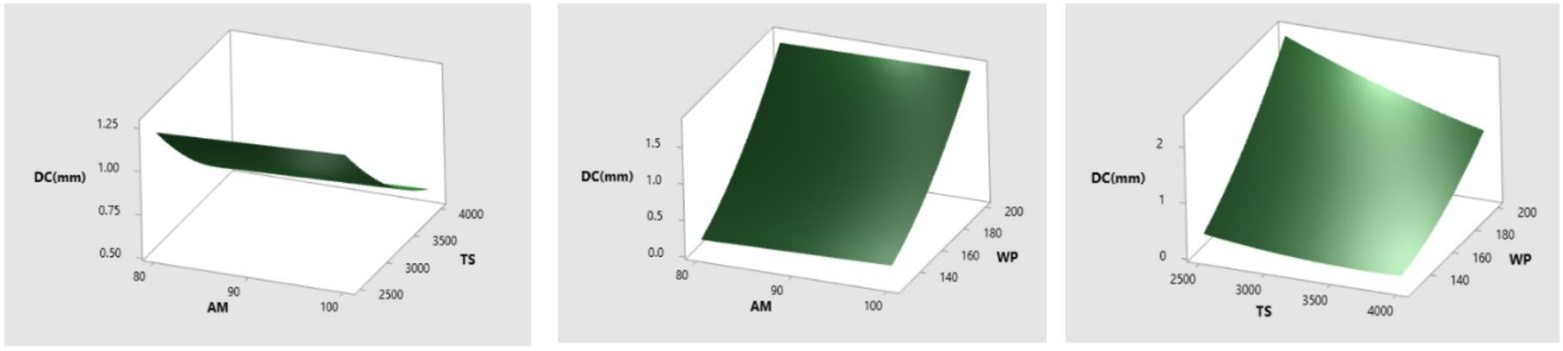
Influence of (**a**) AM&TS (**b**) AM&WP (**c**) TS&WP on DC.

According to the results of the statistical study, the milling parameters that were studied have a considerable impact on the DC of the milled titanium alloy. Because of this, it is extremely important to explore the interaction effects of these factors in AWJ pocket milling operation. Response surface plots are used to demonstrate the effects that the AWJ milling settings have on the DC (Fig. 9). Figure 9(a) shows a response surface plot, which illustrates how the AM and TS have an effect on the DC. Figure 9 clearly demonstrates that there has been a moderate increase in depth from the AM #80 mesh size to the #100 mesh size. This took place because the coarse #80 abrasives collided in the nozzle walls, reducing the energy. Because of the much longer span of time that the jet is exposed to the target material, a greater depth was discovered when the TS was set to a lesser value (2500 mm/min). It is clear from looking at Figs. 9(b) and 9(c) that the depth of the milling pocket gets deeper as the WP value is higher. At high water jet pressures, this phenomenon was seen where there was a large distribution of energy throughout the surface of the target, which created high depth. The WP of 200 MPa produced the greatest amount of depth.

### UC at different AWJ milling parameters

**Fig. 10:**
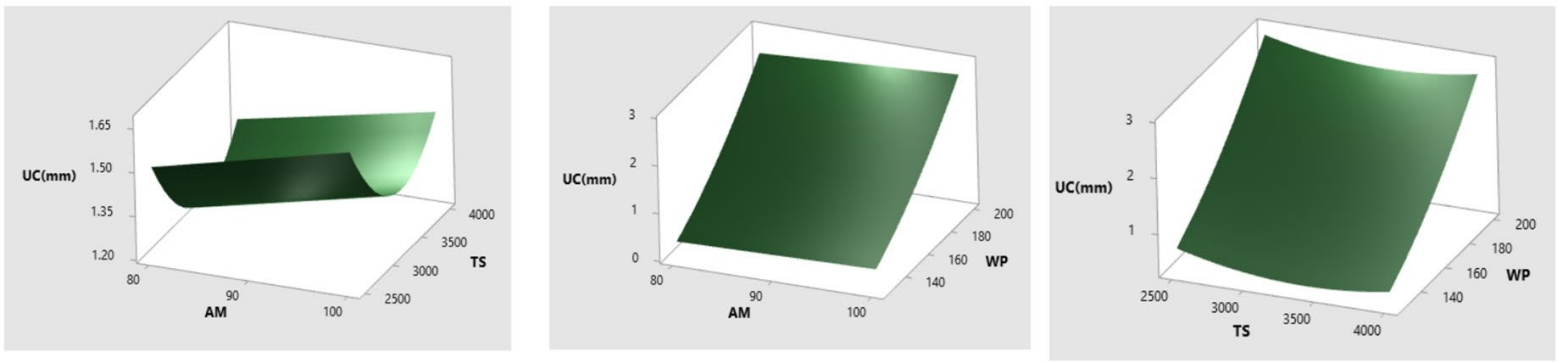
Influence of (**a**) AM&TS (**b**) AM&WP (**c**) TS&WP on UC.

The DC, MRR, and quality of the milled pockets are the primary objectives of the researchers. On the other hand, a few defects were found in pockets milled as UC in the exterior of the pockets. When the raster path enters a corner, the jet may be at zero, resulting in a larger kerf and greater depth milling. Minimal step over distance or dynamic machine settings may induce nozzle speed changes^26^. These flaws were seen in a variety of tool path techniques, including rectangular^4,5^ and triangular^27^ pockets with a change of orientation at 90 degrees. And, only a few research reported that the development of UC occurred in pockets milled using the single slot approach^28^. Because it has such a big impact on average DC, MRR, and surface quality, UC in the milling pockets is the primary factor that affects the overall quality of the components. As a result, it is essential to bring the UC in pocket milling down to an acceptable level. According to the findings of the statistical studies, the AWJ milling settings that were chosen for the experiment had a profound effect on the creation of the UC. Figure 10 illustrates the effects that the AWJ milling settings have on the DC. The impact that AM and TS have had on DC is seen in Fig. 10(a). It can be noticed that the DC is greater at the 100 mesh size at low TS (2500 mm/min). This is due to #100 abrasives experienced less particle collision which kept the energy of the abrasives in the jet stable^29^. As a direct consequence of this, a significant quantity of material was removed from the border region, so producing an UC in that area. However, because the speed of the nozzle slows down when turning a 90-degree angle, this location is also more likely to strike more abrasives than other regions. In addition, the rise in WP and the fall in TS levels both led to a quicker material removal, which resulted in a bigger UC to the titanium alloy, as seen in Fig. 10(c). WP drop and raising the TS to 3500 mm/min decreased the UC depth, after passing this threshold, the UC reached its full potential at 4000 mm/min and 200 MPa WP. Compared to TS values of 3000 and 3500 mm/min, 4000 mm/min decelerated rapidly towards the lowest speed; nonetheless, it was somewhat quicker than the 2500 mm/min nozzle speed and brought in more abrasives in action, which increased UC.

### SR at different AWJ milling parameters

**Fig. 11:**
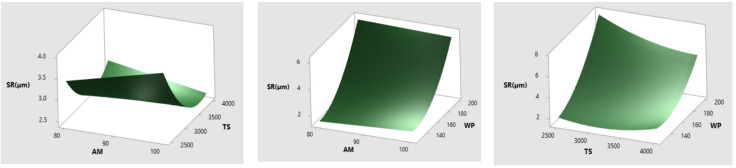
Influence of (**a**) AM&TS (**b**) AM&WP (**c**) TS&WP on SR.

SR is one of the most important quality qualities that should be present in any machined endues components. As a result, the evaluation of the roughness of the milled pockets served as the basis for determining the smooth surface. According to the findings of the statistical studies, the milling parameters that were chosen for the experiment had a huge influence on SR. Figure 11 shows how AWJ pocket milling parameters affect SR. Rough surfaces were discovered, which were the result of significant erosion with increased energy and removal of material in an uneven manner at a WP of 200 MPa (Fig. 11b and c). When the water pressure was raised, the jet stream’s impact energy increased, causing uneven material removal and corrugated surfaces. The water jet’s threshold energy prevented lateral erosion across the target material. This permitted homogeneous flat surfaces at 125 MPa WP, regardless of traverse rate. Greater WP has rough, grooved surfaces. Reduced TS allowed more abrasives to strike sample, resulting in a rough surface (Fig. 11a).

### MRR at different AWJ milling parameters

**Fig. 12:**
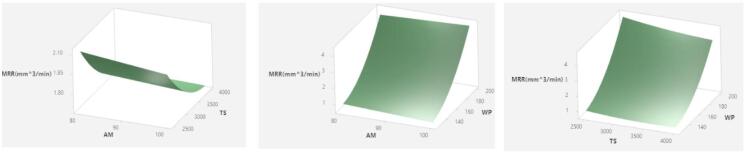
Influence of (**a**) AM&TS (**b**) AM&WP (**c**) TS&WP on MRR.

The MRR has a considerable impact on both the total cost of processing and the rate of output that may be achieved during machining. Because of this, businesses are continuously seeking for methods to enhance their MRR in order to satisfy customers’ needs while keeping their production costs as low as possible. According to the findings of the statistical study, the MRR of titanium alloy is considerably impacted by the process factors that were taken into consideration because the MRR process is dependent on a number of parameters. Response surface plots provide a visual representation of the effects that the AWJ pocket milling settings have on MRR (Fig. 12a–c). It can be observed from the charts that there was a correlation between the increase in WP and the rise in MRR for both of the AM sizes. However, due to the fact that energy was transferred to abrasives from the water jet in the mixing chamber, the MRR was increased by the abrasive mesh size #80 despite the fact that the pressure of the water jet was changed to a lower level. In addition to this, there was a decrease in the TS, which resulted in an increase in the number of abrasives that impinged against the material that was being targeted. Because of the occurrence of these results, the MRR increases because a greater number of abrasives struck the material and removed it. This is because the MRR accounts for the number of abrasives that affected the material. The results reinforce established findings that WP and TS significantly influence key milling outputs such as DC, SR, and MRR, consistent with previous investigations on Ti-6Al-4 V and other alloys. Unlike earlier single-objective studies, our multi-criteria optimization approach integrating advanced evolutionary algorithms combined with CODAS and MOORA multi-criteria decision analysis offers a more holistic and effective parameter selection technique. This integrated approach outperformed conventional studies by balancing multiple performance metrics simultaneously and by efficiently identifying parameter sets that maximize machining quality and productivity.

### Microstructure analysis of machined surface

**Fig. 13:**
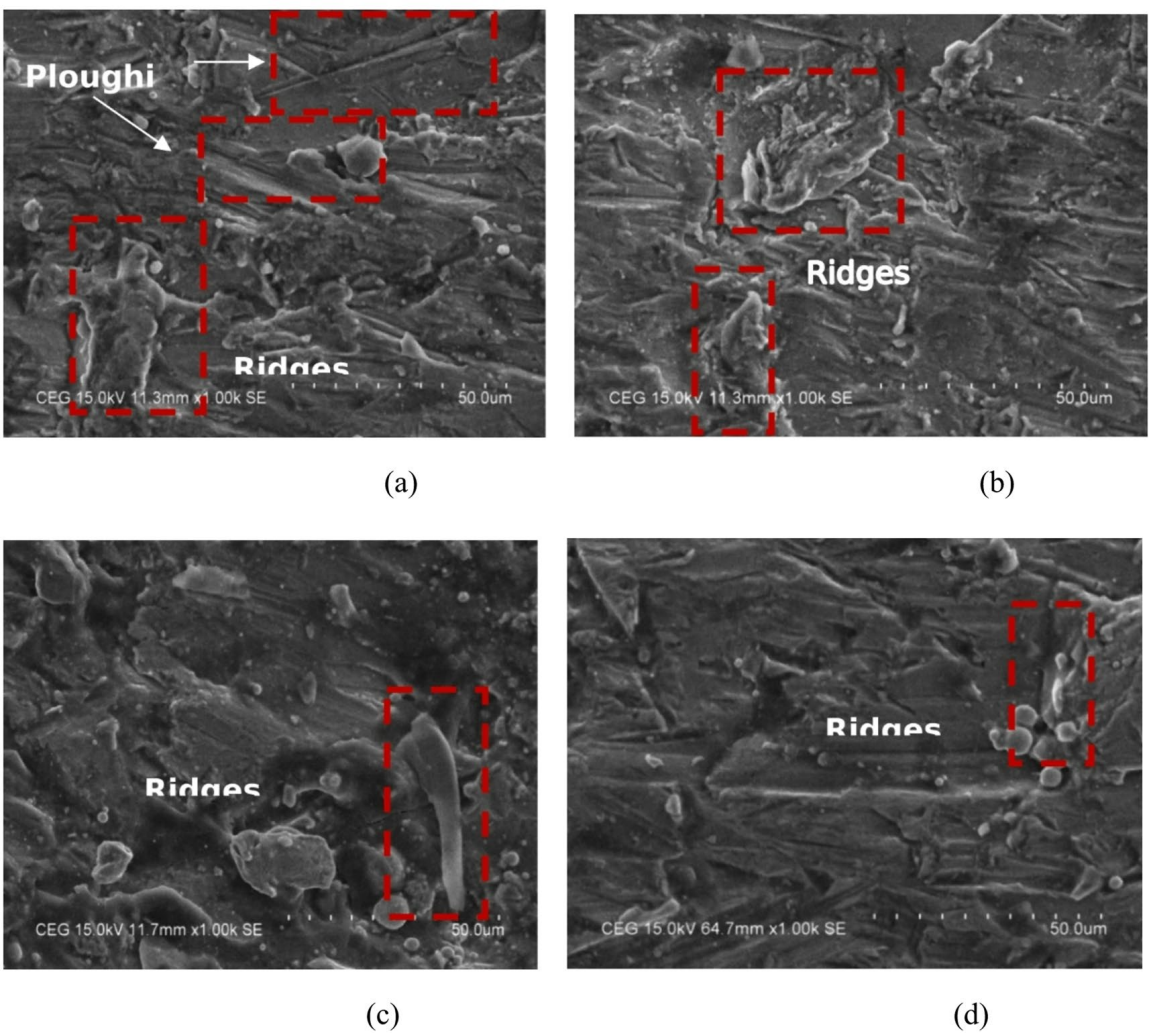
SEM images of #80, #100 milled surfaces at 200 MPa and 2500 mm/min.

The microscopic appearance of milled surfaces is seen in Fig. 13. When comparing mesh size #80 (a&b) milled surfaces to mesh size #100 (c&d) it appears that a greater number of ploughing and deep ploughing were seen. And it was found that surfaces with a mesh size of #100 were the smoothest, with less ridges and wear tracks. It has been established that the shearing action of abrasive mesh size #100 was responsible for the damage, rather than the ploughing impact. While travelling through the mixing tube, the jet’s kinetic energy was gradually decreasing as a result of the intense particle collisions caused by the coarse (#80) abrasives. Because of this, the surface was ploughed as a result of a single abrasive grain being struck with a lesser amount of kinetic energy. Continuous jet action has pushed material to the side of channels, forming ridges. Because of fewer particle collisions and retained cutting energy, #100 milled surfaces have bigger particles than other surfaces. We also found deep gaps due to #100 mesh’s enhanced material removal. In the end, the material from the surface of the target was removed using a mix of operations involving cutting and deformation erosion. To complement the qualitative SEM observations, ridge heights and groove depths were measured directly from the micrographs: average ridge height was approximately 15 μm and average groove depth was about 25 μm. Each SEM image now includes a scale bar to facilitate interpretation.

## Conclusion

In this study, AWJ pocket milling on Ti-6Al-4 V alloy was performed by adjusting the abrasive AM, WP and TS. ANOVA was used to statistically assess the experimental outcomes. To establish the relationship with milling parameters, minitab software was used to create quadratic regression equations for DC, UC, SR, and MRR. Using PSO, MFO, GWO, and WOA algorithms, the optimal combination of milling parameters was projected to maximise DC and MRR while minimising UC and SR. And the performance of the algorithms was compared using the MCDM approach, with the major results shown below.

1. The Analysis shows that WP and TS are important in pocket milling. During milling the jet’s energy depends on the WP and the increase in energy increase the milling depth and as well as material removal rate.
2. Taking into consideration the properties of the pocket was determined that WP was the most influential parameter, followed by TS and AM.
3. SEM micrographs show that the surface of the AWJ milled material had ridges and grooves when it was milled with a high WP.
4. The multi-criteria decision analysis methods such as CODAS and MOORA was introduced to find the effectiveness of the PSO, MFO, GWO and WOA based on the computational time.
5. The minimal values of the performance measurement tool, such as SP and IGD, were compared with the different algorithms and the findings showed that the MFO algorithm obtained less SP and IGD.
6. The optimum combination of milling parameters is obtained from MOORA MCDA method.
7. The MFO algorithm came up with the following values for DC, UC, SR, and MRR: 2.4074 mm, 2.78 mm, 7.6769 μm, and 4.5401 mm/min when the input variables were 80.3210 AM, 2515.9316 mm/min TS, and 200 MPa WP.
8. The optimized parameters identified in this study have practical significance: by maximizing depth of cut and material removal while minimizing undercut and surface roughness, AWJ pocket milling of Ti‑6Al‑4 V can achieve improved surface integrity, reduced machining time and lower abrasive consumption. These improvements translate into cost savings and enhanced component quality, which are particularly valuable for aerospace and biomedical applications. Future work could extend this methodology to other alloys, investigate the effect of different abrasive types and explore hybrid optimization frameworks combining MCDA with advanced metaheuristics.

The optimized parameters obtained in this study are directly applicable to pocket milling operations in aerospace and automotive component manufacturing. In aerospace, they can be used for machining turbine blade slots, cooling channels, and internal cavities of structural components, while in automotive, they are suitable for engine block cavities, transmission housings, and lightweight structural parts. Implementing these optimized settings can result in higher material removal rates, improved surface finish, reduced tool wear, and precise dimensional control, providing measurable improvements in component quality and production efficiency. We acknowledge that the present study considered only two abrasive mesh sizes and a limited pressure range, which may restrict the generalizability of the results. Future research could explore a wider variety of abrasive types and mesh sizes, expanded pressure ranges, and other multi-criteria decision-making (MCDA) frameworks, such as TOPSIS, DENGS. VIKOR, AHP, and PROMETHEE, to further enhance optimization and industrial applicability.

## Data Availability

The datasets used and/or analysed during the current study available from the corresponding author on reasonable request.
